# In vitro propagation for conservation and genetic fidelity of the near threatened *Dimocarpus longan* plant

**DOI:** 10.1186/s43141-022-00406-4

**Published:** 2022-09-07

**Authors:** Manal El-salato Ala El-naby Ahmed

**Affiliations:** Department of Plant Genetic Resources, Desert Research Center, Cairo, 11753 Egypt

**Keywords:** Adventitious buds, Acclimatization, *Dimocarpus longan*, Direct organogenesis, Genetic stability, AFLP, Near threatened

## Abstract

**Background:**

*Dimocarpus longan* is a tropical tree that produces edible fruit. It is a neglected plant species that is listed as near threatened. In spite of its economic value, the propagation of longan cultivar using conventional methods is extremely difficult. The goal of this research is to produce and conserve this plant through in vitro propagation.

**Results:**

In order to form new shoots, sterilized shoot tip explants were cultured on Murashige and Skoog (MS) medium supplemented with benzyl adenine (BA) or 2-isobentenyl-adenine (2ip). For direct organogenesis, young leaves of new shoots were cultured on MS medium fortified with various concentrations of Thidiazuron (TDZ) or 6-(4-Hydroxy-3-methylbut-2-enylamino purine) (Zeatin). Gibbrellic acid (GA_3_) at different levels alone or in combination was used for shoot elongation. Also, indole-3-butyric acid (IBA) and naphthalene acetic acid (NAA) were used for root formation. MS medium supplemented with 1.00 mg/l 2ip was suitable for inducing axillary shoots from shoot tips (4.0 axillary shoots/explant). The highest significant 76% and numbers of adventitious buds from leaf base were achieved on MS medium containing 1.0 mg/l TDZ. These buds developed into the longest plantlets on GA_3_ at 3.0 mg/l and rooted well in ½MS containing 1.50 mg/l IBA plus 0.50 mg/l (NAA). About 70% in vitro plants were successfully acclimatized. The AFLP profile illustrated the genetic stability of gene expression action. The amplified fragment length polymorphisms (AFLPs) profile illustrated the progenies were extremely similar to the mother plants. According to our findings, MS medium containing 25 ppm salicylic acid (SA) and 5 ppm methyl jasmonate (MeJA) produced the highest percentage of apigenin in longan calli (77.09 and 2.637%, w/w).

**Conclusion:**

A successful and efficient micropropagation protocol has been developed and described here for the first time, and it will be very useful for the clonal propagation and conservation of the near-threatened *Dimocarpus longan* plant. Micropropagated plants are genetically identical to the donor plant using the AFLP technique. The usefulness of salicylic acid and methyl jasmonate as elicitors for increasing in vitro production of secondary metabolites in plants is demonstrated in this work.

## Background

Tropical hardwood trees provide sustenance as well as economically useful raw materials such as fodder, fuel wood, timber, and other non-timber items. In ecology, all trees perform an important role [1].

*Dimocarpus longan* Lour., often known as longan or dragon eye fruit, is a tropical and evergreen tree that bears edible fruit. Its fruit is well-liked by people all over the world because of its sweet and juicy taste, as well as its health benefits. It belongs to the Sapindaceae family, which includes *Litchi chinensis* L. (litchi) and *Nephelium lappaceum* L. (rambutan) [2].

Longan fruit has high-vitamin contents, nutrients, phenolic acids, flavonoids, polysaccharides and bioactive compounds that have antimicrobial, antiviral, antioxidant, and anti-inflammatory [3, 4]. Moreover, it has anti-carcinogenic properties, as well as memory-enhancing effects, and has been used as bioactive constituents in folk medicine for a variety of treatments [5, 6] such as improving blood circulation, calming tensions, and alleviating sleeplessness. Longan fruit extract contains three main polyphenolic components: Corilagin, gallic acid, and ellagic acid, found in which are responsible for the antioxidant capabilities [7].

Flavonoids are currently attracting a lot of attention from scientists and the pharmaceutical industry because of their potential health benefits. Flavonoids are also plant secondary metabolites that humans are unable to produce [8]. Antibacterial, antiviral, anti-allergic, antiplatelet, anti-inflammatory, antitumor, and anti-oxidant properties have been reported [9–11].

Apigenin has anti-inflammatory, antioxidant, anti-cancer, neuroprotective, anti-microbial, and anti-allergic properties [12–16]. Apigenin is a flavon that is considered to be safe even at high doses, and no toxicity has been reported thus far. It has been shown in numerous in vitro and in vivo studies to play a key role in the prevention and even treatment of a variety of diseases. Because of its ability to inhibit some of the body’s most important enzymes, this molecule is a promising candidate for use in the treatment and prevention of emerging diseases like diabetes. Modern science has confirmed the traditional use of plants high in apigenin for the treatment of depression and sleep disorders. Furthermore, apigenin has the potential to be used to slow the progression of Alzheimer’s disease [17].

Egypt has a wealth of economically valuable germplasm, particularly in remote far or areas. These germplasms have been threatened by pests and harsh conditions. Rare germplasms in general are a source of riches or fortune in the agricultural field. It is important to pay more attention to the available germplasm in particular, those grown in the arid zone [18].

The use of in vitro culture would give a valuable way of preserving such priceless plants. Rare [19], threatened [20, 21] plant species, and those with low responses to conventional propagation methods have been multiplied and preserved using tissue culture approaches [22].

Longan is an endangered and neglected plant species that is on the verge of extinction [23, 24]. Long reproductive cycle and heterozygous genetic background are limiting factors for longan cultivar conventional propagation. So, micropropagation was used as a suitable way to propagate and prevent genetic erosion of *Dimocarpus longan* [22].

Plant propagation in vitro has many advantages over traditional propagation. Recently, its use in horticulture, agriculture, and forestry is more common around the world*.* In vitro propagated plants are true to type, physiologically uniform, and healthy disease-free plants that can be acclimatized in a shorter period [25].

Micropropagation of tree species provides planting materials for forestation, production of woody biomass, and preservation of superior germplasm [26]. Moreover, in vitro culture of woody plants will play a critical role in plant conservation, propagation, and genetic improvement, as well as restoration ecology [27].

Organogenesis and shoot proliferation in longan are still lacking. Explants removed from adult trees and micropropagated by organogenesis are extremely difficult. In most cases, adventitious buds can be produced, but they perish after culturing. Although adventitious buds have proliferated on occasion, roots remain challenging [2, 28].

Plant growth regulators are important in in vitro medium because they influence cell, tissue, organ, and organ differentiation. Auxins, cytokinins, and gibberellins are all included. These plant growth regulators are defined as organic substances that alter physiological processes to speed up the management of plant growth and other function development [29]. A relationship between a high cytokinin ratio and a low auxin ratio may encourage shoot production, while the opposite supports root formation. Thidiazuron (TDZ) is a derivative phenyl urea (*N*-phenyl-*N*-1, 2, 3-thiadiazol-5-ylurea) that promotes axillary shoot proliferation in a wide range of plant species [30]. TDZ has the ability to overcome apical dominance. It is expected to be more powerful than most commonly used cytokinins, TDZ promotes development directly through biological processes, or it can cause the production and accumulation of an endogenous cytokinin. According to Guo et al. [31], lower concentrations of TDZ are more convenient for axillary shoot formation in woody plant species than higher amounts. BAP and other cytokinins are ineffective in this respect.

It is critical to construct an in vitro propagation system for longan. Many attempts have been undertaken in this area, but only a few successful outcomes have been reported. According to Jiafu and Bizhu [32], 1–3 new buds were regenerated from shoot tips. Longan callus culture was used by Lai et al. [28] to produce somatic embryogenesis. Following that, publications on somatic embryogenesis and plantlet regeneration in longan via pollen culture were published [33]. The current work focuses on developing a micropropagation protocol for *Dimocarpus longan* production. BA and 2iP at different concentrations were used for the multiplication stage. TDZ and zeatin were at different used levels to estimate their effects on organogenesis formation at regenerated leaf bases. GA_3_ and NAA, IBA were used for elongation and rooting stages. Moreover, the genetic differences between the in vitro regenerated plants and the donor plant were assessed using amplified fragment length polymorphisms (AFLP), as well as the production of one of the plant’s main active constituents, apigenin, and increasing its level using different treatments.

## Materials and methods

The current study was undertaken between 2020 and 2022, with the goal of maintaining the longan (*Dimocarpus longan*) through micropropagation.

### Explant collection and sterilization

Shoot tip explants of *Dimocarpus longan* were collected from the adult trees grown at Alkanater Alkhayria, Agricultural Research Center.

Explants were cleaned under running tap water for 2–3 h. After that, explants were sterilized firstly, by using 70% ethanol for 1 min, followed by 20 min in 25% (v/v) Clorox (NaOCl 5.25%), and then washed three times with sterile distilled water to remove Clorox residues. Mercuric chloride at 0.1% (w/v) was used for 5 min as a final sterilization treatment and subsequently washed three times with sterile distilled water in a laminar airflow cabinet.

### Initiation stage

After disinfection, shoot tips (0.5–1-cm long) were planted vertically on Murashige and Skoog’s solid medium [34] (MS). The MS medium was fortified with 30 g/l sucrose and 0.5 mg/l benzyl adenine (BA). Prior the addition of 2.5 g/l gelrite, the medium pH was adjusted to 5.7. The medium was poured into big jars at rate of 40 ml. After that, the jars were sealed with polypropylene covers and sterilized at 121°C for 20 min at 1.12 kg/cm^2^ pressure. Cultured jars were incubated for 16 h at 25±2°C in an air-conditioned incubation room with a light intensity of 3000 lux provided by cool white light fluorescent tubes.

### Multiplication stage

After the initiation stage, the shoots were transferred to the multiplication stage to study the impact of different concentrations of BA and 2-isobentenyl-adenine (2ip) at 0.0, 0.1, 0.25, 0.5, 1.0, and 2.0 mg/l on multiplication rate.

To supply source materials for future studies, the explants were subcultured in glass jars on the optimum medium formula. Survival percentage of explants, the average number of axillary shoots/explant, and shoot length (cm) were measured after 8 weeks.

### Organogensis formation

Leaf explants isolated from in vitro grown shoots were used for direct organogenesis induction and development. The explants were cultured on MS medium supplemented with different concentrations of Thidiazuron (*N*-phenyl-*N*′-1,2,3-thidiazol-5-yl urea) (TDZ) and 6-(4-hydroxy-3-methylbut-2-enylamino purine) Zeatin at 0.0, 0.1, 0.25, 0.50, 1.00, 1.50, and 2.00 mg/l to examine their ability to form organs. Cultures were incubated in total darkness and recultured onto the same fresh medium every 6 weeks. After 14 weeks, the average number of adventitious buds per explant and the frequency of organogenesis were determined.

### Elongation stage

The sprouted shoots were shifted to jars containing full-strength MS medium with different concentrations of gibberellic acid (GA_3_) at 0.0, 1.0, 2.0, and 3.0 mg/l alone or in combination with 2ip 0.5 mg/l in order to obtaining optimum treatment for shoot elongation. Each treatment comprised 10 replicates, and the explants were grown in big jars. The average length of axillary shoots (cm) was recorded after 8 weeks of incubation.

### Rooting stage

In order to form roots, healthy regenerated shoots were placed in half-strength MS basal medium with indole-3-butyric acid (IBA) and naphthalene acetic acid (NAA) at (0.0, 0.1, 0.25, 0.5, 1.0, 1.5, and 2.0 mg/l for each) and 1 g/l activated charcoal (AC). Combination treatments of IBA at 0.5, 1.0, 1.5, and 2.0 mg/l and 0.5 or 1.0 mg/l NAA on root formation were also investigated.

After 6 weeks of cultivation, data on rooting frequency, number of roots, and root length were recorded.

### Acclimatization stage

The in vitro well-rooted plantlets were removed from the cultured jars, washed thoroughly to remove any traces of MS medium, treated with fungicide topsin 20% (w/v) solution, and hardened off in pots filled with a sand-peat moss (1:1 v/v) soil mixture. The pots were covered with transparent polyethylene bags before being placed in a greenhouse.

The covers were gradually removed over the course of a month. The percentage of transplants that survived was recorded.

### Genetic fidelity of in vitro grown plantlets

The genetic differences between the in vitro plants and the donor plant were assessed using amplified fragment length polymorphisms (AFLP) in this study.

### DNA extraction

According to Pirttilä et al. [35], the total DNA was extracted using procedures for medicinal and aromatic plants. RNase A (10 mg/ml, Sigma, USA) was added to the DNA solution and incubated at 37°C for 30 min to eliminate RNA contamination. The concentration of DNA in different samples was calculated by measuring optical density at 260 nm and applying the following equation:

Conc. (ug/ml) = OD260 × 50× dilution factor.

### Amplified fragment length polymorphisms (AFLPs)

AFLP is a technique that uses PCR amplification to detect genomic restriction fragments and can be used on DNA of any origin or complexity. Using a limited set of genetic primers, the fingerprints are created without any prior knowledge of sequence. AFLP allows for the selective amplification of restriction fragments from a total digest of genomic DNA, which is particularly beneficial for detecting variation between the in vitro regenerated plants and the donor plant.

The AFLP technique was used in accordance with Vos et al. [36]. The genomic DNA was cut with two restriction enzymes (EcoRI and MseI) (Table 1) and ligated with double-stranded EcoR 1 and Mse1 adaptors to create the samples. The overhanging sticky ends created by the restriction enzymes were used to ligate the adaptors. On the other hand, the value of genomic template stability (GTS) was calculated for each treatment using the formula GTS% = (1-d/n) ×100 where *d* the average number of bands found in each treatment sample and *n* is the total number of bands in the control sample.

**Table 1:** Sequences of the adapters and the selective primers used in AFLP analysis

Primer adapters	Sequence
***Eco*R1-adapter**	5’-CTCGTAGACTGCGTACC-3’ 5’-AATTGGTACGCAGTCTAC-3’
***Mse*l-adapter**	5’-GACGTGAGTCCTGAG-3’ 5’-TACTCAGGACTCAT-3’
**Primer**	
***Eco*R1**	5’-GACTGCGTACCAATTC-3’
***Mse*1**	5’-GATGAGTCCTGAGTAA-3’

### Chemical feeding

Young leaves of in vitro produced shoots were used as the initial explants to induce callus culture. Leaves were divided into 1 × 1.5 cm segments and placed on MS media containing 1.5 mg/l 2,4-D and 0.5 mg/l Kinetin according to Hassanpour and Niknam [37]. After 4 weeks, the calli were cultured on MS medium supplemented with 1.5 mg/l 2,4-D and 0.5 mg/l Kinetin and augmented with three different concentrations of the two elicitors; salicylic acid (SA) at (0, 25, 50, and 100 ppm) and methyl jasmonate (MeJA) at (5, 10, and 20 ppm), as well as the precursor phenylalanine (Phe) at (25, 50, and 100 ppm), to increase the amount of apigenin and qurecetin.

### Apigenin and qurecetin extraction

The calli were collected after 4 weeks of culture in the dark without disrupting the culture media in the culture vessels. After that, the calli were freeze-dried to achieve dry weight. After that, a kitchen blender was used to grind the dried calli into a fine powder. For each sample, 1g of powdered calli was correctly weighed and ultrasonicated for 15 min, followed by three extractions with 10 ml of ethanol (30 ml total). After filtration, a vacuum rotary evaporator was used to concentrate the mixed ethanolic callus extract, and each residue was balanced to 10 ml using methanol. Each sample was purified *via* a polytetraflage filter prior to HPLC analysis. Each sample was passed through a 0.45-m porosity polytetrafluoroethylene membrane (Nalgene®, New York, USA) before HPLC analysis.

### Determination of apigenin and quercetin by HPLC extraction and determination

The extraction of dried callus was performed by methanol 70%, and determination was according to Biswas et al. [38]. The HPLC system Thermo (Ultimate 3000) consisted of pump, automatic sample injector, and associated DELL-compatible computer supported with Chromelion7 interpretation program. A diode array detector DAD-3000 was used. The Thermo-hypersil reversed phase C18 column 2.5× 30cm was operated at 25°C. Mobile phase consists of distilled water (solvent A) and methanol (solvent B). The UV absorption spectra of the standards as well as the samples were recorded in the range of 230–400 nm. Standard and samples solutions and even the mobile phase were degassed and filtered through a 0.45-μm filter membrane (Millipore).

The compounds were identified by comparing their retention time and UV absorption spectrum of the injected standards.

Inj. Vol: 20 μl

Column: RP- C18

Column size: 2.5× 30cm

Mobile phase: H_2_O: methanol with mixing ratio 75:25

Flow rate: 1.0 ml/min

Temperature: 25°C

Detection: photo diode array (DAD)

### Statistical analysis

Each treatment was repeated three times in the experiments, which were designed up in a totally randomized form. The data were evaluated using analysis of variance and Duncan’s [39] multiple range test (*P* < 0.05).

## Results

### Initiation stage

Shoot tips were found to be good explants since they stayed fresh and green even after being treated with Clorox (NaOCl 5.25%) and HgCl_2_ and had a 70% survival rate, which cultured on MS medium with 0.5 mg/l BA.

### Multiplication stage

#### Influence of various concentrations of BAP and 2ip on growth and development

For the establishment of in vitro cultures, the type of culture medium, source and age of explants, surface disinfection procedures, microbial contamination, and environmental factors are all critical. A review of the literature suggests that any plant regeneration methodology requires careful selection of explants and growth regulators [40]. Preliminary studies for the formation of in vitro shoot cultures using shoot tips were carried out in the current study.

**Fig. 1:**
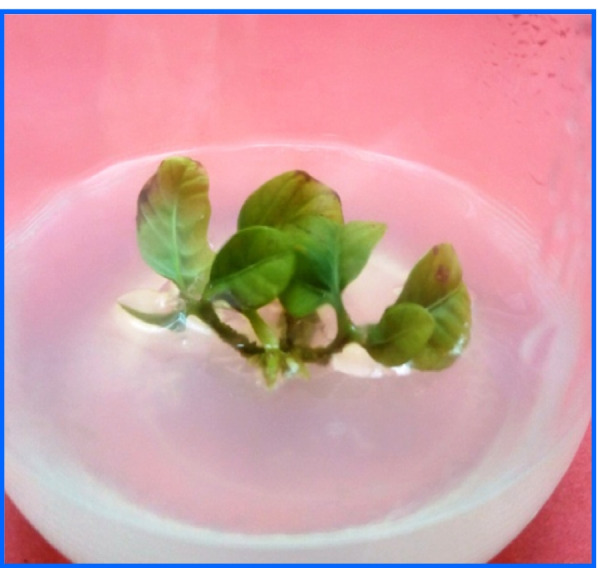
Establishment of *in vitro* shoot tips micropropagation of *Dimocarpus longan*

After 8 weeks of culture, data from the in vitro development of longan shoot tips (Table 2) demonstrated that shoots could be induced with varying degrees on all investigated cytokinin treatments compared with control. Data in Table 2 showed that augmented MS medium with 1.0 mg/l 2ip produced the highest number of shoots (4.0 shoots/explant); however, they were only 1.3 cm long (Fig. 1). The shoot length was considerably higher in MS medium with 0.1 mg/l BAP (2.0 cm). On MS medium containing BAP, however, the frequency of shoot multiplication was rather low (Table 2 and Fig. 1). Previous studies have highlighted the importance of growth regulators in a variety of developmental processes, from germination through the creation of shoots and roots [41].

**Table 2:** Effect of BAP and 2ip on *Dimocarpus longan* growth and development

Growth regulators conc. (mg/l)		Mean number of axillary shoots/explant	Mean length of axillary shoots (cm)	Survival percentage
BA	2ip			
**0.00**	**0.00**	1.0^g^	1.8^c^	40^e^
**0.10**	**0.00**	1.0^g^	2.0^a^	50^d^
**0.25**	**0.00**	2.0^e^	1.9^b^	60^c^
**0.50**	**0.00**	2.0^e^	1.7^d^	60^c^
**1.00**	**0.00**	3.0^b^	1.5^e^	80^a^
**2.00**	**0.00**	2.0^e^	1.4^f^	70^b^
**0.00**	**0.10**	1.7^f^	1.9^b^	30^f^
**0.00**	**0.25**	2.4^d^	1.7^d^	70^b^
**0.00**	**0.50**	2.8^c^	1.5^e^	70^b^
**0.00**	**1.00**	4.0^a^	1.3^g^	80^a^
**0.00**	**2.00**	3.0^b^	1.4^f^	80^a^

Values followed by the same letter in columns are not different at *p* < 0.05 by Duncan’s multiple range test, a = Very highly significant (Very high result), b = Highly significant (High result), c = Significant (Intermediate result), than that d-g mean less significant (Less result), and the same letters were not significant

**Table 3:** Effect of Zeatin and TDZ at different concentrations on shoot regeneration from leaf of *Dimocarpus longan*

Growth regulators conc. (mg/l)		Mean number of adventitious shoots/explant	Organogenesis percentage
Zeatin	TDZ		
**0.00**	**0.00**	0.1^h^	1.00^j^
**0.10**	**0.00**	0.1^h^	1.00^j^
**0.25**	**0.00**	5.5^g^	18.00^i^
**0.50**	**0.00**	9.0^e^	50.00^g^
**1.00**	**0.00**	15.0^b^	70.00^b^
**1.50**	**0.00**	10.0^d^	55.00^ef^
**2.00**	**0.00**	10.0^d^	53.33^f^
**0.00**	**0.10**	0.1^h^	1.00^j^
**0.00**	**0.25**	7.0^f^	25.00^h^
**0.00**	**0.50**	10.0^d^	58.33^cd^
**0.00**	**1.00**	17.0^a^	76.00^a^
**0.00**	**1.50**	11.0^c^	60.00^c^
**0.00**	**2.00**	10.0^d^	56.33^de^

Values followed by the same letter in columns are not different at *p* < 0.05 by Duncan’s multiple range test, a = Very highly significant (Very high result), b = Highly significant (High result), c = Significant (Intermediate result), than that d-j mean less significant (Less result), and the same letters were not significant

#### Organogensis formation: The influence of TDZ and Zeatin concentrations

The in vitro organogenesis of woody species plays an essential role in the improvement of forest products by providing saplings with high commercial value. Direct induction of organogenesis (shoots bud) on explants is a kind of in vitro culture approach that has a number of benefits, including the ability to reduce somaclonal variance, which is prevalent in plants regenerated from callus cells or cell suspension culture. Following that, these protuberances grew and developed into adventitious shoot buds. The presence of younger, actively proliferating cells in that zone may be linked to the induction of shoot buds from the edges of leaf explants [42]. With time, the number of adventitious buds increased, as did their length.

**Fig. 2:**
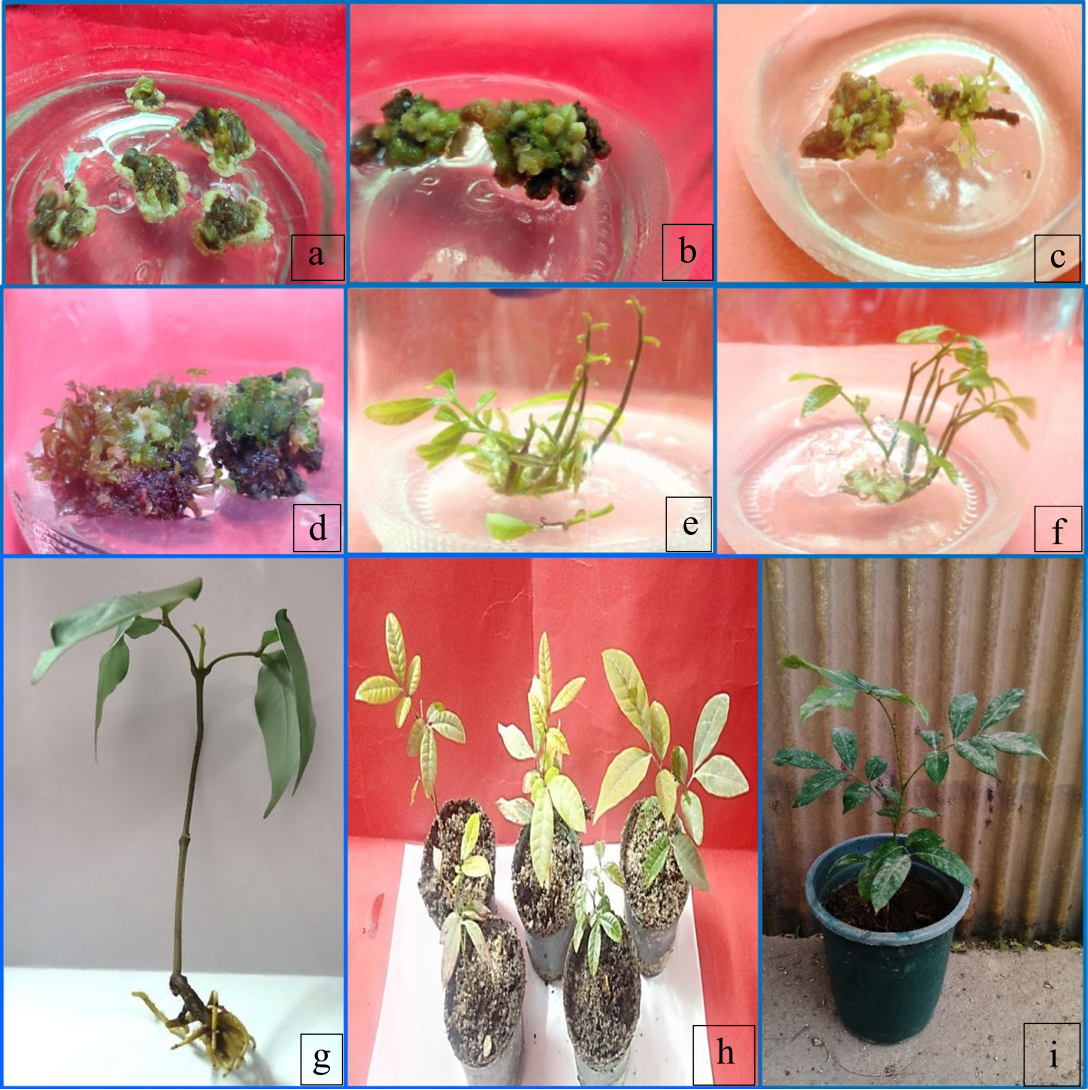
Organonenesis stages of longan (*Dimocarpus longan*) plants. **a** Swelling degree of leaf buds on MS medium containing 1.0 mg/l TDZ. **b** Organogenesis induction from explants cultured on medium containing 1.0 mg/l TDZ. **c**, **d** Organogenesis numbers of longan cultured on medium containing 1.0 mg/l TDZ. **e** Development of organs cultured on medium containing 1.0 mg/l TDZ after 8 weeks. **f** Elongated shoot formation on medium containing 3.0 mg/l GA_3_. **g** Root numbers during rooting stage on MS medium containing 1.5 mg/l IBA and 0.5 mg/l NAA. **h**, **i** Acclimatized plant after 6 and 9 months, respectively

The potential for regeneration from leaf segments using TDZ and Zeatin various concentrations was explored in Table 3. TDZ and Zeatin containing media regenerated new shoots from leaf explants within 14 weeks of incubation in compared with control treatment. The highest percentage of organogenesis formation (76%) and shoot numbers (17) was achieved on medium containing 1.0 mg/l TDZ followed significantly by medium containing 1.0 mg/l Zeatin (70%) and (15) respectively (Fig. 2a–e). Increasing TDZ and zeatin concentrations up to 1.0 mg/l decreased significantly both parameters.

#### Elongation stage: effect of different concentrations of GA_3_ with or without 0.50 mg/l 2ip on elongation of in vitro proliferated shoots

Table 4 shows the effect of various GA_3_ concentrations alone or in combination with 2ip on the elongation of *Dimocarpus longan* in vitro proliferating shoots. In comparison to the other media examined, MS medium with 3.0 mg/l GA_3_ exhibited the longest mean length of shoots (5.6 cm) (Fig. 2f), followed significantly by GA_3_ at the same concentration with 0.5 mg/l 2ip (3.9 cm). Removing GA_3_ from the medium dramatically reduced the length of shoots in the presence and absence of 2ip.

**Table 4:** Effect of different concentrations of GA_3_ with or without 0.50 mg/l 2ip on elongation of *Dimocarpus longan* proliferated shoots

Growth regulators conc. (mg/l)		Mean length of axillary shoots (cm)
GA_**3**_	2ip	
**0.0**	**0.00**	0.5^i^
**1.0**	**0.00**	2.5^g^
**2.0**	**0.00**	3.4^d^
**3.0**	**0.00**	5.6^a^
**4.0**	**0.00**	3.5^c^
**1.0**	**0.50**	2.1^h^
**2.0**	**0.50**	3.2^e^
**3.0**	**0.50**	3.9^b^
**4.0**	**0.50**	2.7^f^

Values followed by the same letter in columns are not different at *p* < 0.05 by Duncan’s multiple range test, a = Very highly significant (Very high result), b = Highly significant (High result), c = Significant (Intermediate result), than that d-i mean less significant (Less result), and the same letters were not significant

#### Rooting stage: effect of various concentrations and combinations of IBA and NAA on rooting of shoots

Inadequate roots is a key stumbling block to plantlet survival in the field and the efficacy of in vitro regeneration protocols [43]. In vitro grown shoots (4–5 cm long) were taken from the culture vessel and planted individually on half-strength MS medium with various concentrations of IBA and NAA. The percentage of shoots that formed roots varied in accordance of type and concentration of auxins (Table 5 and Fig. 2g).

**Table 5:** Effect of various concentrations and combinations of IBA and NAA, on rooting of *Dimocarpus longan* shoots

Auxin conc. (mg/l)		Mean number of roots/explant	Mean length of roots (cm)	Mean shoot height (cm)	Rooting %
IBA	NAA				
**0.00**	**0.0**	0.10^q^	0.10^q^	2.50^m^	1.00^r^
**0.10**	**0.0**	1.00^p^	0.80^n^	3.00^k^	10.00^p^
**0.25**	**0.0**	1.80^m^	1.10^l^	3.40^i^	15.00^o^
**0.50**	**0.0**	2.00^k^	1.50^h^	3.80^g^	20.00^m^
**1.00**	**0.0**	2.50^i^	1.70^f^	3.90^f^	30.00^k^
**1.50**	**0.0**	3.13^g^	1.90^e^	4.00^e^	45.00^g^
**2.00**	**0.0**	3.00^h^	1.60^g^	4.00^e^	40.00^h^
**0.00**	**0.1**	1.30^o^	0.50^p^	2.70^l^	18.00^n^
**0.00**	**0.25**	1.90^l^	0.70^o^	3.00^k^	25.00^l^
**0.00**	**0.50**	2.43^j^	1.00^m^	3.50^h^	30.66^j^
**0.00**	**1.00**	3.00^h^	1.20^k^	4.00^e^	40.00^h^
**0.00**	**1.50**	3.70^e^	1.30^j^	4.20^d^	48.00^e^
**0.00**	**2.00**	3.20^f^	1.1^l^	4.00^e^	45.00^g^
**0.50**	**0.50**	1.00^p^	0.10^q^	3.00^k^	5.00^q^
**1.00**	**0.50**	4.00^d^	2.20^d^	3.10^j^	47.00^f^
**1.50**	**0.50**	6.00^a^	3.00^a^	6.43^a^	69.66^a^
**2.00**	**0.50**	5.00^b^	2.60^b^	3.00^k^	64.66^b^
**0.50**	**1.00**	1.40^n^	2.50^c^	3.50^h^	38.00^i^
**1.00**	**1.00**	1.00^p^	1.40^i^	3.50^h^	30.00^k^
**1.50**	**1.00**	5.00^b^	2.60^b^	5.76^b^	60.00^c^
**2.00**	**1.00**	4.30^c^	2.50^c^	5.00^c^	55.00^d^

Values followed by the same letter in columns are not different at *p* < 0.05 by Duncan’s multiple range test, a = Very highly significant (Very high result), b = Highly significant (High result), c = Significant (Intermediate result), than that d-r mean less significant (Less result), and the same letters were not significant

Within 30 days, root growth was visible at the cut end and nodal region of the shoots, which grew into a large, well-formed root system after 40 days of culture. On medium containing 1.5 mg/l IBA and 0.5 mg/l NAA encouraged the highest root percentage (69.66%), roots number/shoot (6), plantlet length (6.43 cm), and root length (3cm). The lowest significant rooting percentage was appeared on control medium, which was devoid of growth regulators.

### Acclimatization or hardening of plant in greenhouse

After a month of acclimation, the well-rooted plantlets generated fresh new leaves. Approximately 70% of the in vitro plants were successfully acclimatized, with phenotypic similarity to the parent plants (Fig. 2h).

### Identification of AFLP markers

**Fig. 3:**
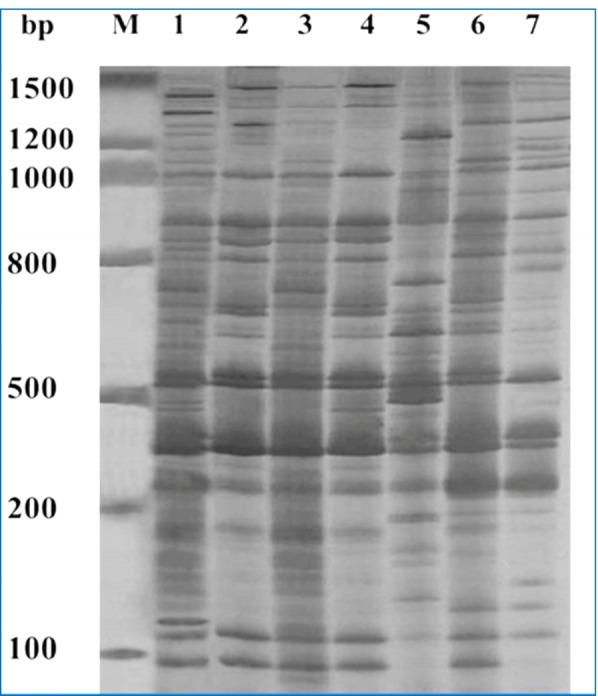
AFLP amplification profile generated from genomic DNA of six *Dimocarpus longan* regenerated plants and its doner plant. M: marker, Lane 1: doner plant. Lane (2–7): in vitro regenerated plants

AFLP analysis with EcoR I-ACA and MseI-CTC primer pairs yielded 95 bands ranging in size from 1648.6 to 101.67 bp (Fig. 3). Thirteen bands were monomorphic bands (1482.6bp, 1670.1bp, 861.9bp, 791.2bp, 709.5bp, 506.9bp, 404.1bp, 308.9, 291.2bp, 249.1bp, 239bp, 117.8bp, and 101.6bp) which reflect the similarity as common bands between all seven samples as shown in Table 6.

**Table 6:** List of specific markers for *Dimocarpus longan* for AFLP marker profile with number of total bands in treatments and their markers molecular weights (MW) in bp

Samples	Total bands	Monomorphic common bands molecular weights (bp)
(1)	37	1482.6bp, 1670.1bp, 861.9bp, 791.2bp, 709.5bp, 506.9bp, 404.1bp, 308.9, 291.2bp, 249.1bp, 239bp, 117.8bp, and 101.6bp
(2)	25	
(3)	30	
(4)	27	
(5)	27	
(6)	26	
(7)	28	

As a result of polymorphism analysis of longan with two primers, the amplification products per profile were described in Table 7.

**Table 7:** AFLP-PCR amplification products of DNA extracted from regenerated plants and its doner plant sample of longan (*Dimocarpus longan*)

Bands	Lane 1	Lane 2	Lane 3	Lane 4	Lane 5	Lane 6	Lane 7
**Mono**	13	13	13	13	13	13	13
**Poly + Uni**	24	12	17	14	14	13	15
**Total bands**	37	25	30	27	27	26	28
**Polymorphism %**	64	48	56	51	51	50	53

The AFLP profile enabled us to discriminate all the samples for studding the genetic stability and illustrated that there are bands appeared in all treatment (common bands), which reflect the genetic stability of gene expression action (Table 8).

**Table 8:** Genomic template stability of six *Dimocarpus longan* regenerated plants and its doner plant according to changes in DNA-AFLP fingerprint

GTS value %						
	Treatment (1)	Treatment (2)	Treatment (3)	Treatment (4)	Treatment (5)	Treatment (6)
**AFLP profile**	33	20	27	27	30	25

### Apigenin and quercetin leveles in Calli

Flavonoids have a variety of therapeutic properties, including anticancer, antioxidant, anti-inflammatory, and antiviral effects. They are also neuro- and cardio-protective. The type of flavonoid, its (possible) mode of action, and bioavailability all influence these biological activities. These low-cost medicinal ingredients have significant biological activities, and their efficacy for a variety of diseases has been demonstrated [44].

Apigenin and qurecetin content varied between the two elicitors and precursor levels. Data presented in Table 9 reveal that two HPLC flavonoids, apigenin and quercetin, were detected after 30 days post-elicitation and precurarization of longan calli. Harvested callus cultures were able to accumulate both apigenin and quercetin.

**Table 9:** Apigenin and quercitin content as an active compounds identified by HPLC in *Dimocarpus longan* callus treated by biochemical elicitors and precursor

Treatments (ppm)	Apigenin content by HPLC mg/g dr.wt	Increase (fold)	Quercitin content by HPLC mg/g dr.wt
**Mother plant (derived from tissue culture)**	3178.584^a^	0.00^j^	N.D^h^
**Leaf callus (control)**	13.204^j^	0.00^j^	N.D^h^
**25 SA**	1018.067^b^	77.09^a^	N.D^h^
**50 SA**	31.420^d^	2.379^c^	18.512^a^
**100 SA**	23.227^e^	1.75^d^	4.091^g^
**5 MeJA**	34.830^c^	2.637^b^	N.D^h^
**10 MeJA**	22.357^f^	1.69^e^	11.252^c^
**20 MeJA**	16.413^h^	1.24^g^	12.025^b^
**25 Phe**	10.692^k^	0.80^i^	7.857^e^
**50 Phe**	14.698^i^	1.11^h^	5.227^f^
**100 Phe**	21.645^g^	1.63^f^	9.076^d^

*N.D.* not detected

Values followed by the same letter in columns are not different at *p* < 0.05 by Duncan’s multiple range test, a = Very highly significant (Very high result), b = Highly significant (High result), c = Significant (Intermediate result), than that d-k mean less significant (Less result), and the same letters were not significant

The results showed that among all treated callus cultures, the highest apigenin content detected with 25 ppm SA and 5 ppm MeJA (1018.067 and 34.830 mg/g dr. wt, respectively), followed by SA elicitation treatment at concentrations of 50 ppm (31.420 mg/g dr. wt), compared with control treatment that recorded 13.2048 mg/g dr. wt.

After 30 days of callus elicitation with SA at concentrations of 50 ppm, the quercetin content was accumulated significantly (18.5120 mg/g dr. wt) more than all the other treatments including SA, MeJA, and control.

**Fig. 4:**
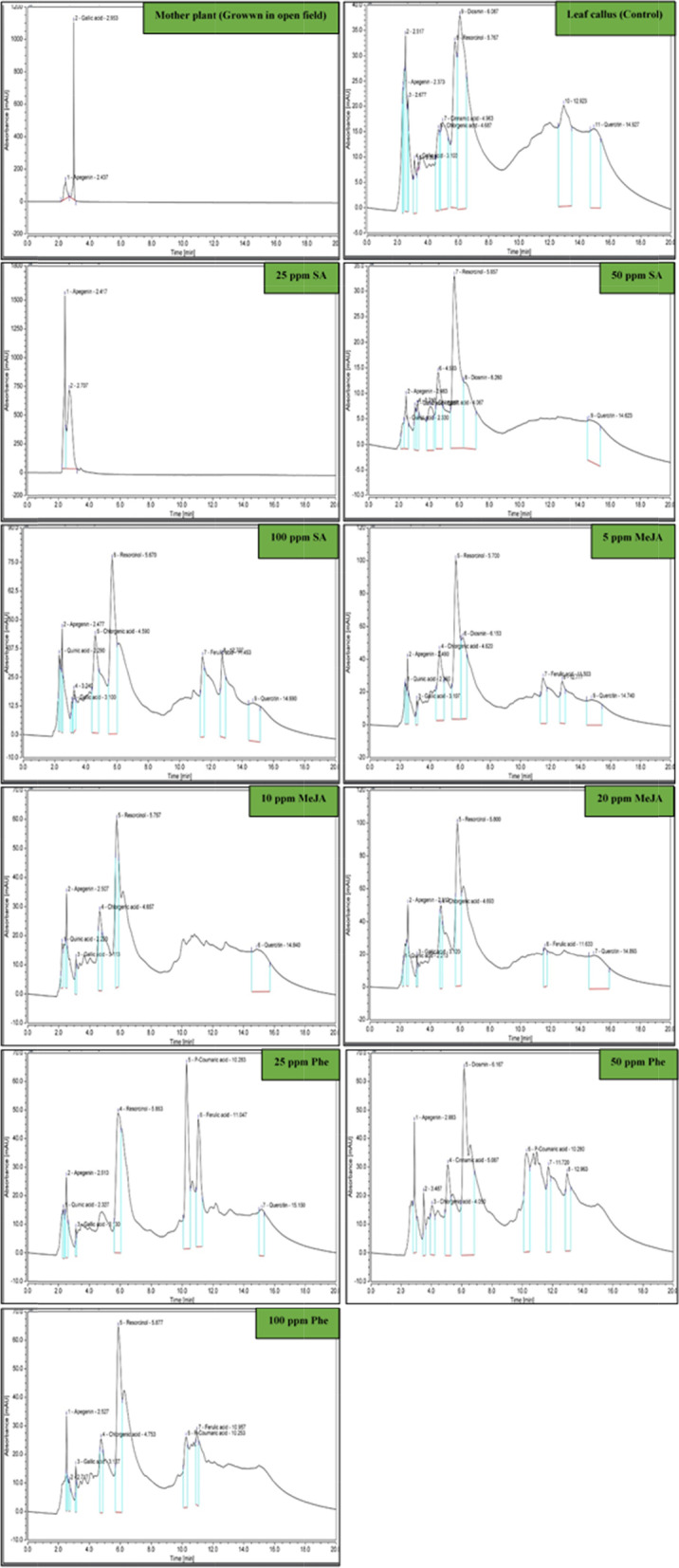
HPLC chromatogram of apigenin and quercitin content in *Dimocarpus longan* callus treated by biochemical elicitors and precursor. Mother plant (grown in open field); callus culture without additives (control); callus culture with 25 ppm salicylic acid (SA); callus culture with 50 ppm SA; callus culture with 100 ppm SA; callus culture with 5 ppm methyl jasmonate (MeJA); callus culture with 10 ppm MeJA; callus culture with 20 ppm MeJA; callus culture with 25 ppm phenylalanine (Phe); callus culture with 50 ppm. Phe; callus culture with 100 ppm Phe

Elicitation with SA at concentration of 25 and 50 ppm, slightly increased accumulation level of both apigenin and quercetin in calli cultures, compared to control calli (Fig. 4).

## Discussion

Recent improvements in woody tree micropropagation have opened up new possibilities for mass multiplication of important genotypes [45]. Explant selection, nutritional media composition, plant growth regulators concentrations, and micropropagation methods all had a substantial impact on longan explant shoot multiplication and rooting rates.

Shoot tips explants were successfully established in vitro on MS medium. One of the most extensively used basal media in tissue culture is MS medium. In vitro culture, according to Ritchie and Hodges [46], medium composition is a critical factor. To ensure the growth of the explants, the medium should contain macro- and micro-elements, vitamins, and the necessary growth regulators.

Multiplication of axillary shoots was influenced by cytokinin type and concentration. The MS medium supplemented with 2ip at 1.0 mg/l resulted in the significantly higher mean number of axillary shoots. In the present study, 2ip was more effective in the production of multiple axillary shoots than BA. This confirms the importance of using 2ip in the multplication medium, which is in harmony with the results obtained by Mendoza et al. [47] found that MS medium containing 2.0 mg/l 2ip produced the highest multiplication efficiency of *Gaultheria pumila* plants. Also, Karyanti et al. [48] who found that 2ip and BAP were more suitable for shoot, root, and leave formation of *Zingiber officinale* in vitro culture compared with the other cytokinins and the highest multiplication rate was noticed on medium containing 2ip. Moreover, Kulpa et al. [49] reported that 2ip has a positive impact on plant development of *Thymus vulgar* during multiplication stage. Furthermore, 2ip was found to be the most effective cytokinin for *Rhododendron indicum* shoot elongation and node development [50]. Otherwise, MS medium with 0.3 mg/l BA + 0.2 IAA+ 0.5 mg/l GA_3_ was the best medium for improving bud formation of longan shoot tip explants with 1–3 new buds/explant through 30–50 days [32]. Furthermore, for the other plant species, *Phyllanthus urinaria* and *Phyllanthus tenellus*, the combination of acytokinins and auxins has been shown to induce shoot bud production in vitro [51, 52].

Direct organogenesis formation from young leaf was cultured on MS medium containing different concentrations of TDZ and Zeatin. MS medium supplemented with 1.0 mg/l TDZ was the best treatment, producing the highest percentage of organogenesis formation of 76% and the highest mean number of shoots/explant of 17. This result is in harmony with that obtained by De Carvalho et al. [53] who found that TDZ-induced direct organogenesis from leaf explants of *Passiflora miniata*. In the present study, TDZ was more effective in the production of organogenesis from leaf than Zeatin. TDZ is a prospective phenylurea (*N*-phenyl-1,2,3-thidiazol-5-yl urea) alternative that has huge potential as a cytokinin in shoot multiplication in a variety of plant systems, particularly in woody species [54, 55]. Taha et al. [56] also found that TDZ was superior for direct organogenesis in three date palm cultivars.

Many scientists have tried to find out how TDZ works in plants. Dey et al. [57] believed that TDZ causes cells in the apical meristem to divide and multiply, then develop, resulting in bud differentiation. Mundhara and Rashid [58] mentioned that calcium stress triggers TDZ ability to drive shoot bud formation in the dark and subsequently affect ethylene production. The metabolism of endogenous growth regulators is strongly linked to the activity of TDZ in morphogenesis. Furthermore, TDZ administration raised endogenous auxin, ethylene, and ABA levels [59].

Cytokinin is the most significant plant hormone for promoting cell division and differentiation, as well as plant growth and development [60]. The physiological role of cytokinin, according to Kulaeva [61], is to activate RNA, protein synthesis, and enzyme activity. Different forms of cytokinin could be used to create many shoots by breaking the apical shoot’s dominance [62].

Length of shoots depended upon the phytohormones type and concentration. In this study, the longest shoots (5.6cm) were obtained on medium contaning 3.0 mg/l GA_3_. This results agree with the results obtained by Suarez Padrón et al. [63] discovered that increasing GA_3_ in the media caused *Alpinia purpurata* shoots to grow longer. Also, Ali et al. [64] and Brondani et al. [65] reported that gibberellins are openly utilized in vitro to boost length in micropropagated shoots in order to improve plant survival when transferred to ex vitro settings and to improve plant performance. Plant growth regulators are chemicals that affect plant growth and development in extremely small levels [66]. Gibberellins are plant growth regulators that promote cell division and elongation, as well as plant organ growth and development [67]. GA_3_ is one of the active plant growth regulators (PGR) for plant growth in tissue culture. In addition to its involvement in germination, this chemical helps to replace the need for light and warmth for growth. GA_3_ stimulates stem elongation (in shoots), size expansion on flower and leaf, leaf color change, aging inhibition, and processing on plant organs [68].

Root formation is a difficult step in the in vitro propagation of many woody plants. Rooting of woody plants is usually induced by auxin. In the present study, two auxins (IBA and NAA) were tested for root formation, because of their low oxidative rate and high stability in the plant in vitro culture. In particular, IBA is more stable than NAA; therefore, it is the most widely used auxin for root induction [69]. The optimum medium for rooting of axillary shoots of *Dimocarpus longan* was half-strength MS medium supplemented with 1.5 mg/l IBA in combination with 0.5 mg/l NAA. It gave 69.66% rooting with the highest mean number and length of roots per explant and mean length of axillary shoots. These results obtained from the current study are close to the results of Jiafu and Bizhu [32] who found that longan buds grew into plantlets and rooted in ½ MS + 0.5 mg IBA/l after 3–4 weeks. In *Phyllanthus amarus*, a similar result was found [70].

Rooting experiment was carried out in this study using two auxins combined, as well as some of the treatments separately with other literature such as Shiragave [71] who reported that the efficiency of IBA than other auxins in rooting in *Phyllanthus reticulatus*. Also, *Paulownia tomentosa* and *Paulownia fortuneii* had the longest root and density when Saiju et al. [72] employed MS medium with NAA at (0.5 or 1.5 mg/l) in the growing medium. Furthermore, the number of almond peach rootstock hybrids has grown on MS medium containing IBA [73].

The interaction of exogenous and endogenous auxin concentrations in the medium and cultured shoots, as well as their uptake, transport, and metabolism, could explain the differential in response [43]. The number of roots is considered an important factor for enhancing the survival of plants during acclimatization and is a sign of a qualitative rooting response [74]. In the present study, approximately 70% of the in vitro plants were successfully acclimatized.

The genetic fidelity and true-to-type nature of the in vitro regenerated plants were validated by monomorphism in the banding pattern acquired using AFLP markers, suggesting that the established methodology for micropropagation of *Dimocarpus longan* was adequate.

On the molecular genetics level, bands of DNA markers were effectively used to identify significant molecular markers and adequate distinctions among the seven samples of which were complement with other chemical analysis data and succeed to have great relevance.

This observation agrees with that of Mirzaei et al. [75] concluded that true-to-typeness of micropropagated olive was cultivar-dependent using partec flow cytometry (FCM) and AFLP analysis. Also, Wójcik et al. [76] employed two DNA-based approaches to assess genetic stability of micropropagated plants, amplified fragment length polymorphism (AFLP) and inter simple sequence repeat (ISSR), and found no polymorphism among the analyzed gooseberry cultivars. Other plant species with consistent genetic fidelity in in vitro regenerates include *Prunus ulcis* [77], *Brassica oleracea* [78], and *Rhodiola imbricate* [79].

According to Karp et al. [80], the AFLP technique is quickly becoming the tool of choice for evaluating genetic activity in both cultivated and treatments. AFLP markers were also used by Neqi et al. [81] to investigate inter- and intraspecific genetic variations in some species of an important wild medicinal plant. They concluded that AFLP markers are an effective tool for estimating genetic effective analysis, as they reflect the genetics expression of transcription and translation rate during the action of some genes pathways.

The first step in creating high levels of bioactive metabolites is to optimize callus induction culture. In vitro production of secondary compounds in medicinal plants is influenced by the types and concentrations of elicitors and precursors [82].

SA is a plant signal molecule that causes changes in plant metabolism at various levels in response to environmental stressors. Salicylic acid elicited growth-promoting responses in mice. SA increased the manufacturing of phenylpropanoid pathway defense chemicals, resulting in a buildup of coumarin-related substrates [83].

Our findings show that MS medium containing 25 ppm SA and 5 ppm MeJA produced the highest percentage of bioactive compounds (apigenin) (77.09 and 2.637%, respectively, w/w) in longan calli, and that it can be used for cell suspension culture. The presence of apigenin in longan has not been previously reported in wild and in vitro plants.

Salicylic acid at the concentration of 50 ppm gave the highest quercitin accumulation (18.5120 mg/g dr.wt).

Our results are in line with Srivastava and Gupta [84] who found that the content of apigenin [0.74% (w/w)] and apigenin-7-glucoside [1.11% (w/w)] in wild *M. chamomilla* aerial parts. Plant growth regulators, on the other hand, have been shown to affect the production of secondary compounds in callus tissues in some studies. The apigenin content of *M*. *chamomilla* in Iran ranged between 0.74 and 1.11% [85].

## Conclusion

This is the first report of effective micropropagation protocol of *Dimocarpus longan* leaf explants by direct organogenesis. TDZ appeared to be more suitable growth regulators especially at 1mg/l than Zeatin for organogenesis formation. Incorporation of GA_3_ in culture medium stimulated shoot elongation. The addition of 1.5 mg/l IBA + 0.5 mg/l NAA to rooting medium encouraged the highest values of all parameters during rooting stage. Micropropagated plants are genetically identical to the donor plant using AFLP technique. This approach could be used to propagate and conserve this commercially important plant species. Our findings show that MS medium containing 25 ppm SA and 5 ppm MeJA produced the highest percentage of bioactive compounds (apigenin) (77.09 and 2.637% respectively, w/w) in longan calli. The presence of apigenin in longan has not been previously reported in wild and in vitro plants.

## Data Availability

All data generated or analyzed during this study are included in this published article.

## Abbreviations

AFLP: Amplified fragment length polymorphism
ANOVA: Analysis of variance
BA: 6-Benzyl adenine
GA_3_: Gibberellic acid
IBA: Iindole-3-butyric acid
2iP: N6-(2-isopentenyl) adenine
IUCN: International Union for Conservation of Nature
Kin: Kinetin
MeJA: Methyl jasmonate
MS: Murashige and Skoog
NAA: α--Naphthaleneacetic acid
Phe: Phenylalanine
PGRs: Plant growth regulators
SA: Salicylic acid
TDZ: Thidiazuron
